# Intergroup Encounters Among Wild White-Faced Capuchins (*Cebus imitator*) at a Densely Populated Field Site: Insights into Frequency, Intensity, and Participation

**DOI:** 10.1007/s10764-026-00548-z

**Published:** 2026-04-06

**Authors:** Sarah A. Kovalaskas, Agostina Colosimo, Vasco Alexandre Martins, Juan Carlos Ordoñez, Elena Morera Saborío, Jacinta C. Beehner, Thore J. Bergman, Marcela E. Benítez

**Affiliations:** 1 Department of Anthropology, Emory University, Atlanta, GA USA; 2 Capuchins de Taboga, Guanacaste, Costa Rica; 3 University of Portsmouth, Portsmouth, UK; 4 Department of Anthropology, University of Michigan, Ann Arbor, MI USA; 5 Department of Psychology, University of Michigan, Ann Arbor, MI USA; 6 Department of Ecology & Evolutionary Biology, University of Michigan, Ann Arbor, MI USA; 7 Department of Biology, Utah State University, 5305 Old Main Hill BNR117A, Logan, UT 84322 USA

**Keywords:** Intergroup competition, Territoriality, Capuchin monkey, Cooperation, Population density

## Abstract

Intergroup encounters (IGEs) are a major feature of social life for group-living primates, influencing access to resources, reproductive opportunities, and territory. We examined IGE frequency, intensity, and participation in a high-density population of wild white-faced capuchin monkeys (*Cebus imitator*) at the Capuchinos de Taboga research project in Guanacaste, Costa Rica, using 4 years of data on three capuchin groups (4,984 observation hours, 218 IGEs). The IGE rates at Taboga (0.044/hr) were two to four times higher than those at nearby lower-density sites and did not vary with rainfall or temperature. More than half of IGEs involved high-intensity aggression (chases or contact aggression). Ordinal logistic models showed that the number of focal group participants was a consistent predictor of escalation, with each additional participant increasing the odds of higher intensity by 1.4 times, while ecological variables had little explanatory value. Participation was more likely for older individuals, males, alphas, and when individuals were closer to the center of core use areas; males participated more than females at distances further from the center of core use areas. Females participated in 44% of IGEs, with lactating females less likely to participate than cycling or pregnant females. Our findings suggest that high population density and overlapping ranges drive frequent and aggressive IGEs at Taboga and that escalation is shaped primarily by social rather than ecological factors. These results highlight the importance of social processes in territorial defense and underscore their role in shaping intergroup conflict in areas of high population density.

**Supplementary Information:**

The online version contains supplementary material available at 10.1007/s10764-026-00548-z.

## Introduction

In social animals, intergroup competition is a critical component of group living, shaping the survival and fitness of group members (Crofoot & Wrangham, 2010). The outcome of intergroup encounters (IGEs)—or social interactions between conspecifics from two distinct groups— can provide immediate and long-term fitness benefits, such as acquiring and maintaining access to key resources, such as food, territory, or mates (Fedigan & Jack, 2004; Fashing, 2001; Williams *et al.*, 2004). Intergroup competition can also have devastating consequences for group members such as injury or death for participants (Mitani *et al.*, 2010; Scarry & Tujague, 2012), loss of territory (Fashing, 2001; Mitani *et al.*, 2010; Williams *et al.*, 2004), disease transmission between groups (Ryu *et al.*, 2020), and nutritional or social stress from consistent outgroup threat that ultimately impacts offspring survival and fitness outcomes (Lemoine *et al.*, 2020). This balance of costs and benefits makes IGEs a central focus for understanding group dynamics and individual decision-making in social animals.

Intergroup encounters occur across a wide range of taxa, from social insects to large mammals. Honey ants (*Myrmecocystus mimicus*), for example, engage in highly organized conflicts to defend territory and resources from neighboring groups (Lumsden & Hölldobler, 1983). Similarly, lions (*Panthera leo*) display cooperative territorial defense, with participation often determined by group size and composition (Heinsohn & Packer, 1995) and among spotted dolphins (*Stenella frontalis*) group synchrony impacts the outcomes of intergroup competition (Cusick & Herzing, 2014). In primates, IGEs are frequently shaped by complex social and ecological dynamics, with patterns of aggression varying across species (reviewed in Majolo *et al.*, 2020). For example, chimpanzees (*Pan troglodytes*) are known for territorial patrols that can escalate into lethal aggression (Mitani *et al.*, 2010), while species, such as bonobos (*Pan paniscus*), rely more on affiliative behaviors to diffuse tensions during encounters (Furuichi, 2011). Even within species, social and ecological variables—such as resource availability, group size, and group composition—play critical roles in shaping the dynamics of these encounters (Furuichi, 2020; Lucchesi *et al.*, 2020). Intergroup encounters are expected to escalate towards aggression when there is strong competition for resources (Sterck *et al.*, 1997) and when the resource holding potential (RHP), or the fighting ability, of groups are evenly matched (Cassidy *et al.*, 2015; Crofoot *et al.*, 2008; Green *et al.*, 2021; Martínez-Íñigo *et al.*, 2023).

An important concept in the animal contest literature related to RHP is resource value (Kokko, 2013). In territorial contests, areas of more intense space use are often interpreted as reflecting greater resource value, as repeated use indicates consistent access to key resources. In high-density populations, compression of home ranges may increase overlap of intensively used areas, potentially increasing the frequency and intensity of IGEs. For example, studies on Kanyawara blue monkeys (*Cercopithecus mitis*) (Butynski, 1990) and Yakushima Japanese macaques (*Macaca fuscata yakui*) (Sugiura *et al.*, 2000) show increased IGE frequency and intensity in high-density populations. In contrast, low-density populations, such as Ngogo blue monkeys, exhibit fewer or no IGEs due to increased home range sizes and reduced competition for resources (Mitani & Rodman, 1979). Similar density-driven patterns are evident in Diana monkeys (*Cercopithecus diana*), where higher densities correlate with stronger or more frequent territorial aggression (Decellieres *et al.*, 2021).

Population density can also shape an individual’s decisions to participate in IGEs due to heightened risk. Several factors, including age, sex, rank, and reproductive status, influence the likelihood of participating in IGEs. In general, when group interests are more aligned, as in single-male, multifemale groups, participation is more predictable. However, in multimale, multifemale groups, males and females often employ distinct strategies during intergroup competition (Beehner & Kitchen, 2007). Male primates commonly engage in IGEs to defend access to mates and prevent outgroup male immigration (Fashing, 2001; Majolo *et al.*, 2005). In contrast, females are more likely to participate when critical resources for offspring survival, such as food and water, are at stake (Arseneau-Robar *et al.*, 2016). In densely populated areas, where competition for resources is heightened, the potential costs of not engaging—or the benefits of successful resource defense may be amplified, intensifying selective pressures on females to participate in intergroup conflicts. This pattern is evident in female colobus monkeys, who participate more aggressively in IGEs as population density increases (Arseneau-Robar *et al.*, 2023).

White-faced capuchin monkeys (*Cebus imitator*) live in multimale, multifemale groups characterized by female philopatry and male dispersal (Fragaszy *et al.*, 2004). Capuchins exhibit remarkable social complexity, including cooperating with group members during coalition formation, group territory defense, and predator defense (Crofoot *et al.*, 2011; De Aquino *et al.*, 2022; Perry *et al.*, 2003); however, intergroup interactions are often hostile and can lead to severe injury or death (Gros-Louis *et al.*, 2003; Scarry & Tujague, 2012). Within their groups, individuals navigate competing interests, with males engaging in IGEs to assess neighboring group composition and secure future mating opportunities, while females defend critical resources like food and water (Crofoot, 2007; Perry, 1996; Schoof & Jack, 2013). Given their highly social nature and the potential for IGEs to escalate into severe aggression, capuchin monkeys represent an excellent model for examining the factors that influence when IGEs occur, why some escalate into aggression, and which individuals participate.

A growing body of research has documented the occurrence and characteristics of IGEs in capuchin monkeys. At Lomas Barbudal, IGEs in one monkey group occurred once every 84 daylight hours (approximately one per week) and increased slightly during the dry season (Perry, 1996). In Santa Rosa National Park, a highly seasonal dry forest, IGEs happen more frequently—around once every 40 daylight hours—and tend to rise in the dry season (Schoof & Jack, 2013; Table I). The intensity of IGEs across capuchins is highly variable, ranging from vocal threats to lethal contact aggression (Fedigan & Jack, 2004; Perry, 1996). Although in capuchins intergroup aggression is not typically tied to immediately defendable resources (Mitchell, 1989; Perry, 1996), IGEs are thought to play an important role in longer-term processes, such as territorial defense and access to mates (Scarry, 2012). Work at capuchin field sites has also highlighted the potential importance of spatial context—such as proximity to core areas of the home range and access to water sources—in shaping where and when between-group interactions occur, particularly in seasonally dry forests (Campos & Fedigan, 2009; Perry, 1996; Schoof & Jack, 2013). However, systematic evaluations of how these spatial and ecological factors relate to variation in IGE intensity remain limited.

**Table I:** Intergroup encounter (IGE) rates and population density at three white-faced capuchin sites

Sites	No. IGEs	Observation hours	IGE rate (no. IGEs/ob hr)	Capuchin density (individuals/km^2^)	IGEs source	
**Lomas Barbudal**	44	3703	0.01	5.84		Perry (1996)
* Wet Season*	-	-	0.10			
* Dry Season*	-	-	0.13			
**Santa Rosa**	33	1198	0.027	11.66		Schoof & Jack (2013)
* Wet Season*	6	257	0.023			
* Dry Season*	27	941	0.028			
**Taboga**	218	4984.25	**0.044**	36.24		Kovalaskas *et al*. (this publication)
* Wet Season*	114	2397.92	**0.048**			
* Dry Season*	104	2586.32	**0.040**			

Observation hours refer to total hours spent monitoring focal groups of wild white-faced capuchins (*Cebus imitator*). Rate is calculated as IGEs per observation hour. Population densities recorded from Tinsley Johnson *et al*. (2020)

Social factors are also expected to play a role in determining IGE intensity. Male white-faced capuchins are more likely to participate in IGEs and behave more aggressively when supported by other group members (Meunier *et al.*, 2012), suggesting that social facilitation and audience effects can promote recruitment and escalation. At the same time, smaller groups successfully win encounters when conflicts occur near the core of their home range, indicating that local familiarity and coordinated participation can offset numerical disadvantages (Crofoot *et al.*, 2008). Together, these findings suggest that under conditions of high density and compressed space use, IGE intensity may be driven less by absolute power asymmetries and more by social influence, coordination, and context-dependent advantages tied to space use. While systematic quantification of IGE intensity in other capuchin populations is limited, descriptions of capuchin IGEs at Lomas suggest that direct aggression during IGEs are relatively rare (Perry, 1996).

Individual participation in IGEs among white-faced capuchins is likely influenced by a range of demographic (e.g., age, sex, rank), ecological (e.g., seasonality, distance to key resources), and social (e.g., group composition) factors. Males—particularly alpha males—are typically the most frequent participants in capuchin IGEs, as they have a strong incentive to maintain dominance, secure mating opportunities, and protect offspring from infanticide (Brasington *et al.*, 2017; Crofoot, 2007; Perry, 1996). In contrast, females participate less often in IGEs in white-faced capuchins. At Lomas Barbudal, for example, females took part in only five of 44 documented encounters (Perry, 1996). However, recent findings suggest that under high-density conditions, female primates may escalate their involvement in IGEs, particularly when maternal energetic stress coincides with resource scarcity (Arseneau-Robar *et al.*, 2023; Lewis *et al.*, 2020). In addition, female participation may vary with reproductive state, as the costs and benefits of engaging in IGEs differ for cycling, pregnant, and lactating females (Scarry, 2012). The ways in which these factors interact to influence participation patterns and intensity levels in high-density capuchin populations are not yet well characterized.

The Taboga Forest Reserve, a 516-ha tropical dry forest home to the Capuchinos de Taboga research project (hereafter Taboga), is located in the northwestern Guanacaste province of Costa Rica and has one of the highest recorded densities of white-faced capuchin monkeys (*Cebus imitator*) (Tinsley Johnson *et al.*, 2020). Taboga encompasses a fragmented habitat shaped by anthropogenic disturbance including agricultural activities, and artificial water sources. Home to approximately 12 capuchin groups—three of which have been fully habituated since 2017—the density of capuchins at this site is two to six times higher than that observed at other field sites (Tinsley Johnson *et al.*, 2020). Taboga supports high capuchin population densities with its abundant resources, including agricultural crops such as sugar cane and a diverse array of native plant species. However, human activities at the site and forest fragmentation have compressed available habitat, reducing home-range size and increasing territorial overlap. These high-density conditions magnify both the likelihood of IGEs and the stakes of those conflicts. This combination of high population density and rich resource availability provides a contrast to nearby lower-density capuchin sites, such as Lomas Barbudal and Santa Rosa, where communities likely travel further, inhabit larger territories, and experience greater ecological stress (Jacobson *et al.*, 2024; Beehner *et al.*, 2022).

In the current study, we address three questions concerning IGEs in this high-density population:

1. How frequently do IGEs occur at Taboga, and how do these rates compare to those reported at nearby lower-density sites?
2. What factors are associated with variation in the intensity of aggression observed (e.g., threats, displays, chases, contact aggression) during IGEs?
3. Which characteristics (demographic, ecological, social) predict individual participation in IGEs?

Given the limited theoretical and empirical work addressing the drivers of IGE intensity and participation in capuchins in relation to population density, our analyses addressing these questions are exploratory in nature. In addition, we conduct a focused sub-analysis examining how female reproductive state relates to participation in IGEs, providing further insight into the costs and benefits of engagement for females under high-density conditions.

## Methods

### Study Site and Subjects

We conducted this study at the Capuchinos de Taboga research project, in the Guanacaste province of Costa Rica (Tinsley Johnson *et al.*, 2020) and collected the data for this study across 4 years (May 2018–May 2022) on three groups of wild white-faced capuchins that are under near daily observation by the Capuchinos de Taboga field team. During this study period, group composition shifted slightly across all three groups such that Tenori ranged from 14–21 individuals (4–6 adult females, 1–3 adult males), Mesas from 13–16 individuals (2–5 adult females, 1–2 adult males), and Palmas from 35–40 individuals (5–7 adult females, 4–5 adult males). In addition to these three study groups, we collected census and demographic data on two additional groups that are often involved in IGEs, Ramas, who split from Palmas in 2021, and Eskameca, which was not fully habituated at the time but has been followed on multiple occasions with all adult animals identified. At the south end of the reserve, Tenori, Palmas, and Ramas have overlapping home ranges, and to the north, Mesas and Eskameca have overlapping home ranges. In addition to these five groups, capuchins occasionally interact with other recognized nonstudy groups in the reserve, identified by territory use and distinctive individuals but not yet fully habituated.

The Taboga Forest Reserve experiences two distinct seasons: a hot, dry season lasting from late November to April and a cooler, wet season from May to early November. The dry season is characterized by a mean daily maximum temperature of 35.38 ± 0.20 °C (SE) and mean daily rainfall of 0.66 ± 0.27 mm (SE), while the wet season exhibits a mean daily maximum temperature of 32.57 ± 0.21 °C (SE) and mean daily rainfall of 8.93 ± 1.09 mm (SE) (Tinsley Johnson *et al.*, 2020). We defined seasonal transitions in May and November using rainfall data (mm) as the start or end of ≥4 consecutive days of consistent rain for comparison with other sites. We recorded the following rainy season dates: May 12–November 8, 2018; May 9–November 18, 2019; May 19–November 28, 2020; May 24–November 7, 2021; and starting April 28 in 2022.

### Data Collection

*Ranging and behavioral data*. We monitored one to two groups at a time each week, from the time they left their sleeping site in the morning (~5:30 hr) until they settled into a sleeping site for the evening (~18:00 hr). We collected ranging data during daily group follows by recording the geographic location of the group every 30 s using a handheld Global Positioning System (GPS) navigator (Garmin eTrex20-22), allowing us to determine the distance each group travelled per day, the home range and core areas, and the locations of all IGEs. As part of routine monitoring for all groups, we record observation hours, and all individuals present as part of the daily census.

*Demographic variables*. In terms of demographic factors that can influence participation in IGEs, we focused on age, alpha status (yes/no) for males and females, and the reproductive state of adult females. For all individuals born before October 2017, or new individuals who migrated into a group, we estimated dates of birth based on body size and morphological characteristics. Age estimations were conducted by experienced Costa Rican field biologists (J.C.O. and Alex Fuentes), who combined, have more than 40 years of experience habituating and studying white-faced capuchins in the region. For all individuals born after October 2017, we recorded their date of birth within 1 week of observation of the new infant. In our models, we represented age as a continuous variable for each individual, determined from known or estimated dates of birth. When we included age classes in analyses of group composition, individuals were categorized as follows: infants (0–2.0 years), juveniles (2.0–6.0 years), subadult males (6.0–10.0 years), and adults. We classified males as adults at ≥10.1 years, and females as adults at their first conception or at 6 years of age, whichever occurred first.

For all adult females, we designated three reproductive stages retroactively based on the median gestation length and interbirth intervals (IBI) known for capuchin monkeys (Fedigan *et al.*, 2008; Perry, 2012). We considered females “Pregnant” at any time within 160 days before the birth of the infant (Carnegie *et al.*, 2011). We considered females “Lactating” starting at infant birth and continuing for the next 456 days based on a median IBI of 2 years (and subtracting gestation time and 3 months of cycling), unless the infant died or the next birth occurred in this time period, from which we would again calculate the pregnancy period. We assigned “Cycling” status when a female was neither pregnant nor lactating, or 456 days after infant birth.

Typically, alpha males are the most active participants in capuchin IGEs at other sites (Perry 1996; Rose & Fedigan 1995), with lower ranking male participation showing more variability. At Taboga, like other capuchin field sites, unambiguous dominance hierarchies (for both males and females) are difficult to discern because antagonistic interactions between dyads are rare (Fragaszy *et al.* 2004; Perry 1998; Schoof & Jack 2013). However, clear patterns of dominance interactions and receipt of submissive signals consistently identified the top-ranking male and female, allowing us to assign alpha status (yes/no) through expert assessment (J.C.O.).

*IGEs.* Across the 4-year study period, we observed 218 IGEs across three focal groups, yielding a rate of 0.044 IGEs per hour (218 IGEs/4,984.25 hours of daily observation). For all IGEs between capuchin groups, we collected data on the location (e.g., GPS point at start and end of encounter), duration (start/end time), demographic information for both the focal and rival groups, number of individuals participating (e.g., “participants”), identity of participants, behaviors and vocalizations, and the intensity of the encounter (e.g., threats, chases, and contact aggression). We initiated the intergroup protocol if any individuals in our capuchin group behaved in a manner consistent with hearing or seeing another capuchin group. These behaviors included sudden changes in movement, such as descending from the trees, moving silently and quickly, travelling terrestrially, sniffing the ground, or splitting suddenly from the group. At the start of an IGE, we recorded the time and a GPS point at the beginning of the change in behavior. If the group split into two parties, one researcher followed the “participants” advancing towards the other group while the other researcher remained behind with the non-participants.

During an IGE, we used recording devices to dictate key pieces of information such as the identity of the other group (if known), the number and identities of the individuals participating in the encounter, the number of other group individuals participating, and any specific interactions that we observed between animals, including vocalizations, displays, and aggressive behaviors. Following previous definitions of participation in capuchin IGEs, individuals were deemed “participants” in the encounter if they engaged in a social interaction with a monkey from another group or moved forward towards the opposing group and followed out-group monkeys, as opposed to remaining stationary or fleeing in the opposite direction (Van Belle & Scarry, 2015). For each IGE in which we verified the presence of another group (e.g., group seen or heard by researchers), we assigned one of four intensity levels based on observed behaviors:

1. We saw or heard a group but groups did not escalate to any form of aggression;
2. We observed displays or threats between groups;
3. We observed chases between groups, and
4. We observed contact aggression between groups.

**Fig. 1:**
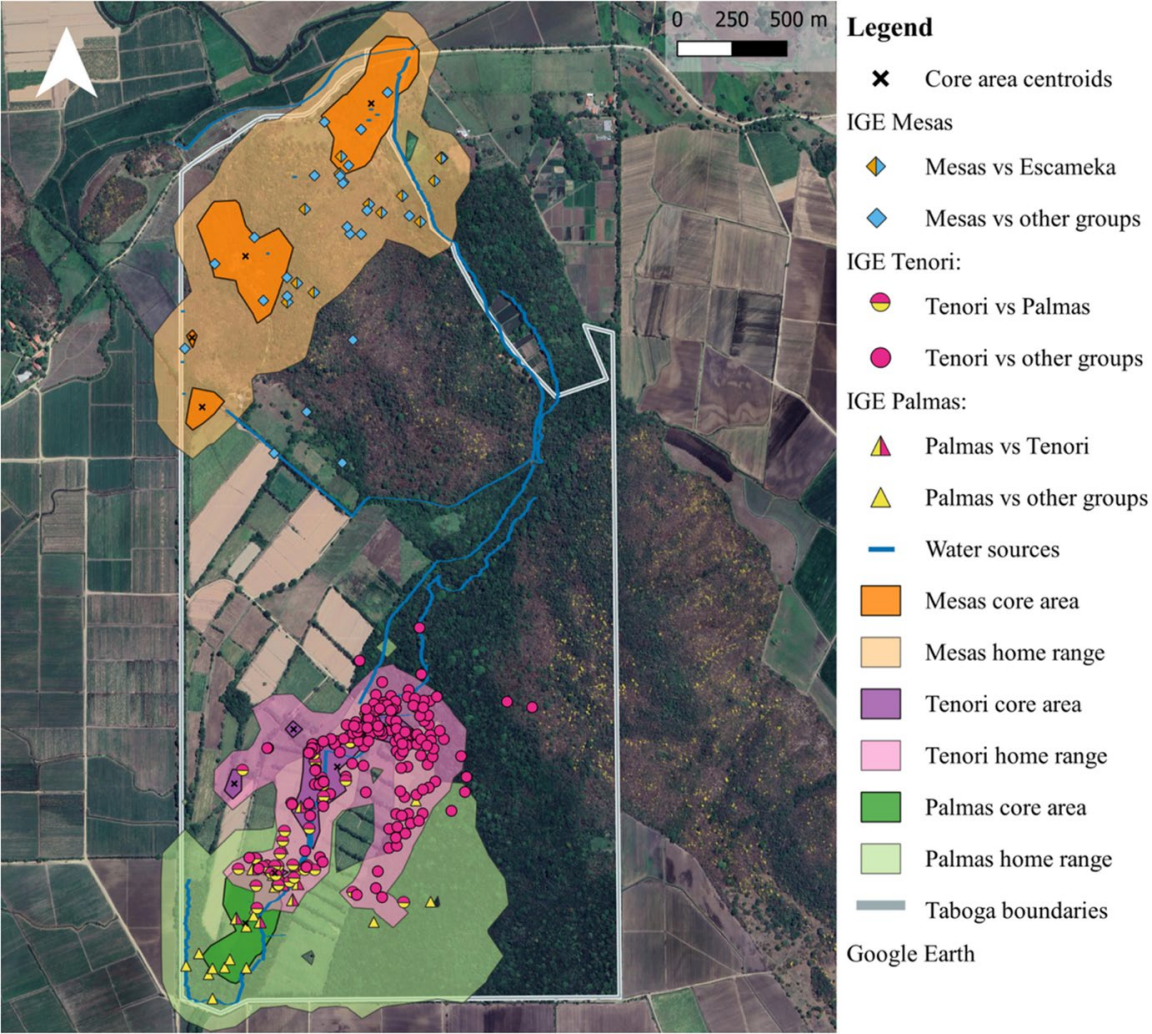
**Home range area (95% kernel density estimation) and core areas (50% kernel density estimation) of three groups of wild white-faced capuchins (*Cebus imitator*) at Taboga, Guanacaste, Costa Rica (May 2018–May 2022).** The home ranges of Tenori and Palmas overlapped over an area of 0.324 km^2^, representing 43.7% of the Tenori home range and 27.2% of the Palmas home range. Spatial data are displayed over high-resolution satellite imagery.

*Home range and core areas.* Using ranging data, we conducted analyses in R version 4.1.2 (R Core Team, 2021) using the package *adehabitatHR* (v. 0.4.21; Calenge, 2011). We differentiated *home range area* (95% kernel density estimation) and *core areas* (50% kernel density estimation) of the Tenori, Mesas, and Palmas capuchin groups with GPS data collected during the study period and visualized these areas using QGIS software (v. 3.16.16; QGIS Development Team, 2021). Distance from the center of the home range is an important variable shaping the outcome of IGEs in white-faced capuchins (Crofoot *et al.*, 2008). For this reason, for every IGE observed, we used the GPS point taken at the start of the IGE to calculate the distance from the nearest core area centroid of the focal group’s home range (*dist.centroid*) (Fig. 1) and the nearest water source to investigate how these variables impacted intensity of (question 2) and participation in (question 3) IGEs (Fig. 1).

## Ethical Note

The data collected for this study were noninvasive behavioral observations that complied with the legal requirements of Costa Rica, with permits obtained from el Sistema Nacional de Áreas de Conservación (SINAC) (# M-P-SINAC-PNI-ACAT-005–2021). All protocols involved in this study were previously approved by Emory University IACUC (no. PROTO202100033). The authors declare that they have no competing interests.

### **Data Availability**

Data are available upon reasonable request to the authors. Data are not publicly deposited currently due to ongoing analyses.

### Statistical Analyses

*Frequency of IGEs at Taboga.* We conducted all the following statistical analyses in R (version 4.4.0; R Core Team, 2024). First, we examined the overall frequency of IGEs at Taboga by dividing the total number of IGEs observed by the total number of contact hours spent observing all groups. Next, we investigated whether seasonal environmental factors, such as cumulative monthly rainfall and maximum monthly temperatures, influenced the rate of IGEs. Given that our data followed a negative binomial distribution, we tested two statistical models—a general linear mixed model (GLMM) with a Poisson distribution and a negative binomial regression—to assess how our environmental predictor variables, monthly cumulative rainfall and maximum temperature, impacted the frequency of IGEs. We implemented models using the *lme4* (v. 1.1.35.3) and *glmmTMB* (v. 1.1.9) packages in R (Bates *et al.*, 2015; Brooks *et al.*, 2017). In both models, we included scaled total monthly rainfall and scaled maximum monthly temperature as fixed effects, and group as a random effect. The response variable was the count of IGEs per month for each group, offset by the number of contact hours (log-transformed) to account for differences in observation effort. To determine which model was a better fit, we compared Akaike Information Criterion (AIC) values and tested for overdispersion using a Pearson chi-squared test. According to AIC values, the models were almost identical in fit (Poisson: 292.9 vs. NegBinomial: 294.9) however the Poisson model had fewer parameters and no overdispersion (dispersion ratio 0.844, χ^2^ = 68.40, *p* = 0.84). Furthermore, we detected no temporal autocorrelation (using ACFs of Pearson residuals within groups across months), thus we determined the Poisson model to be the best fitting and most parsimonious model for our analysis. We assessed statistical significance for the Poisson GLMM assessing IGE frequency with Wald z-tests implemented in *lme4* for glmer objects.

*Intensity of IGEs at Taboga.* Second, we examined how social and ecological factors impacted the intensity of an IGE. For social factors, we examined whether the number of participants from the focal group influenced the intensity of an IGE. For ecological variables we examined cumulative rainfall (mm, previous 10 days), high daily temperature (°C), distance of IGE to the centroid of the nearest core area (m), and distance to the nearest water source (m). In addition, we examined whether the duration of the IGE (min) was related to the overall intensity of that encounter. We scaled continuous predictors (e.g., cumulative rainfall, temperature, distances) to standardize their ranges, improve model interpretability, and ensure that all predictors contributed evenly to the model. Due to limited dyadic replication across group pairings, we omitted group-level random effects to ensure model stability and avoid over-parameterization. To check for multicollinearity among predictors, we calculated the Variance Inflation Factor (VIF) using *vif()* from the *car* package in R (Fox *et al.*, 2013). All VIF values were below 1.2, indicating that multicollinearity was not a concern.

We used a series of ordinal logistic regressions to assess the impact of social and ecological factors on the intensity of IGEs rather than a single global model owing to sample size limitations and concerns about over-parameterization. We constructed the models using the *polr()* function from the *MASS* (v. 7.3.60.2) package (Venables & Ripley, 2002) in R. For our ordinal logistic regression models, the proportional-odds assumption was evaluated using a Brant test (*Brant* v. 0.3.0; Schlegel & Steenbergen, 2020) and was not violated. As an initial screening step, we evaluated each predictor in a single-predictor model relative to a null (intercept-only) model to gauge support and guide construction of a limited multivariable candidate set. We then fit multivariable candidate models centered on the most supported predictor(s), including models that added spatial and ecological covariates (distance to core, distance to water, temperature, rainfall) and encounter duration. For model selection, we used the *bbmle* (v.1.0.25.1) (Bolker & R Development Core Team, 2025) package in R and compared the corrected Akaike Information Criterion (AICc) (n = 190 observations, with complete information for all variables), which adjusts for small sample sizes and relative support was assessed using Akaike weights. The final candidate models evaluated for IGE intensity are summarized in the Supplementary Information (S3).

*Participation in IGEs at Taboga*. Third, we examined the impact of demographic and socio-ecological factors on an individual capuchin’s likelihood to participate in an IGE. For each IGE, any individual present in the group was considered a potential participant. We classified individuals as participants in the IGE if they engaged in any direct interaction, such as threats, displays, chases, or aggression, with members of the opposing group or moved towards the other group after detection. Thus, for each IGE, an individual received a “yes” if they participated and a “no” if they were present and did not participate. 

We considered the following predictors in relation to whether an individual participated (yes/no): 1) individual demographics (age, sex, alpha status); 2) ecological factors (cumulative rainfall (mm), high temperature, distance of IGE to the nearest water source (m), and distance of the IGE to the centroid of the nearest core area (m); and 3) group composition (group size, number of adult females, number of adult males, and number of infants). To control for repeated sampling of individuals, we included a random effect for individual ID. We did not include group-level random effects due to limited dyadic replication in group pairings and to avoid over parametrization. In all models, we scaled continuous predictors (e.g., age, high temperature, rainfall, and distances) to facilitate interpretation of coefficients. We constructed multiple generalized linear mixed models (GLMMs) using the *glmer()* function from the *lme4* package. Given the binary nature of the response variable, we fit logistic regression models with a binomial error distribution and logit link function. We compared candidate models compared using Akaike’s Information Criterion corrected for small sample sizes (AICc) and relative support was assessed using Akaike weights. We calculated AICc values and model weights using the *bbmle* (v.1.0.25.1) (Bolker & R Development Core Team, 2025) package, and inference was based on relative model support rather than selection of a single best model.

As an exploratory step, we first evaluated single-predictor screening models within conceptual categories (individual demographics, ecological/spatial variables, and group composition) to identify variables with explanatory signal and to identify predictors that reduced sample size due to missing data. We then constructed a limited set of multivariable candidate models that combined the most supported predictors across categories and included interaction terms only when biologically relevant (e.g., sex × distance to the home-range centroid). The final candidate models evaluated for participation are summarized in the Supplementary Information (S4). We extracted effect sizes and confidence intervals for supported models from fitted model objects and reported these alongside model selection results for the best supported models.

Because reproductive stage is expected to impact the likelihood of female participation in IGEs, we conducted a separate analysis focused specifically on adult females with data from 164 IGEs. These females represented adults for which we had comprehensive data pertaining to life history events. To determine the impact of reproductive stage on whether a female participated in an IGE, we employed a mixed-effects binomial logistic regression with a dependent variable of participation in an IGE (yes/no) using *lme4* (v. 1.1.35.3). In this model, we included the predictor variable reproductive stage (pregnant/lactating/cycling), with individual ID as a random effect. To further explore differences in the levels not directly compared in the original mixed-effects binomial logistic regression predicting female participation in an IGE (*yes/no*), we conducted *post hoc* Tukey tests on relevant predictors Table II.

**Table II:** Outcome and predictor variables for four analyses: 1) Frequency of IGEs; 2) Intensity of IGEs; 3) Participation in IGEs; 4) Female participation in IGEs of wild white-faced capuchins (*Cebus imitator*) at Taboga, Guanacaste, Costa Rica (May 2018-May 2022)

Outcome variables	Predictors
**IGE rates** # of IGEs per month (per group)	Monthly rainfall (mm) Max temperature (C)
**Intensity level**	Cumulative rainfall (previous 10 days) Max temperature Distance of IGE to the centroid of the nearest core area (m) Distance to nearest water source (m) IGE duration (min) # of participants from focal group
**Participation (All)**	Individual demographics age sex alpha (yes/no) Ecological factors cumulative rainfall (mm) high temperature distance of IGE to nearest water source (m) distance of IGE to the centroid of the nearest core area (m) Group composition group size # of adult females # of adult males # of infants Sex* Rainfall Sex*Dist to Core
**Participation (females)**	Cycling Pregnant Lactating

## Results

### Frequency of IGEs at Taboga

**Fig. 2:**
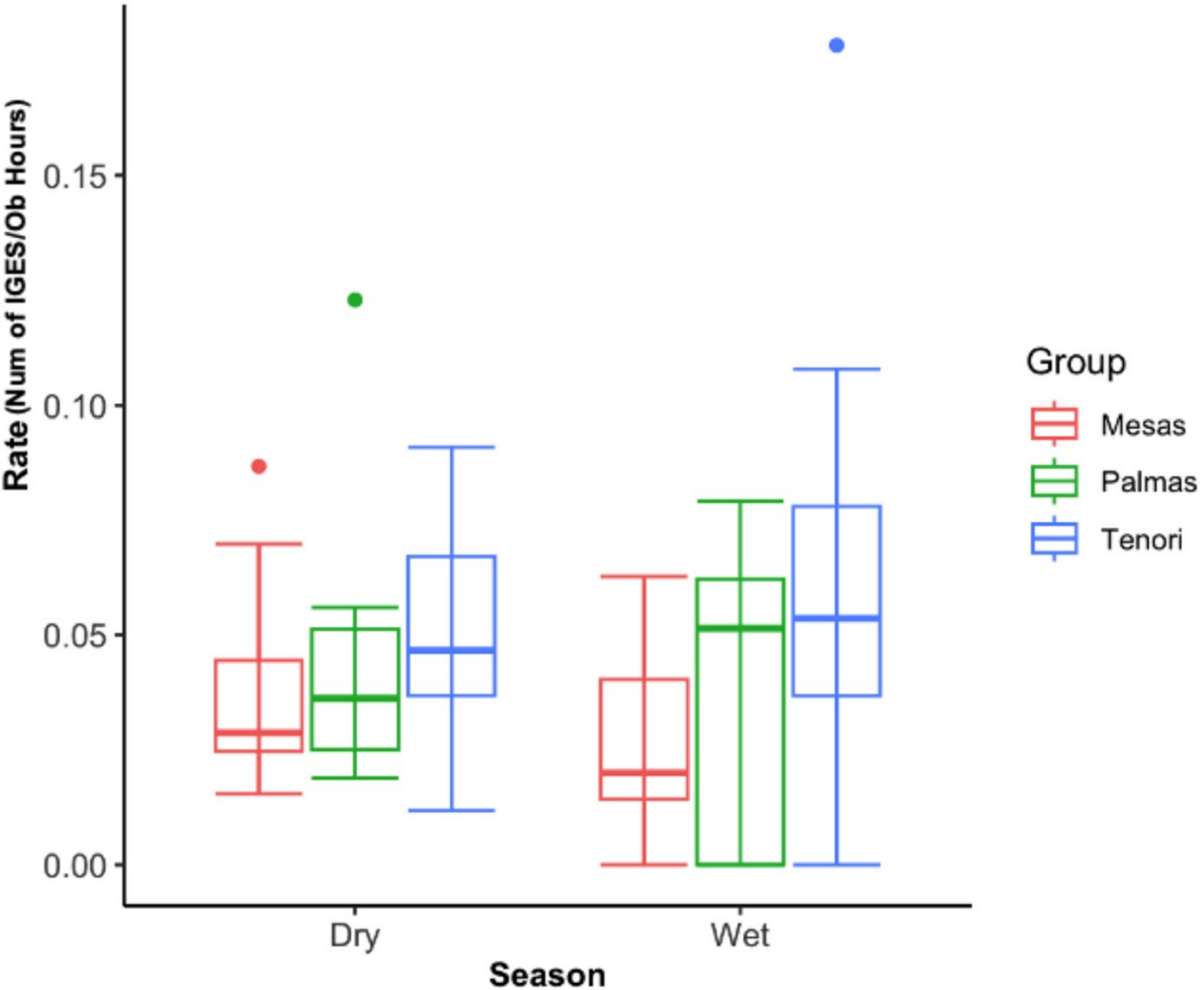
**Rates of IGEs at by season across three main study groups (Mesas, Palmas, and Tenori) of wild white-faced capuchins (*Cebus imitator*) at Taboga, Guanacaste, Costa Rica (May 2018-May 2022).** Boxes indicate the interquartile range (IQR) of encounter rates, (IQR), whiskers extend to values within 1.5 × IQR, horizontal lines denote medians, and points indicate outlier values.

We found no significant seasonal effects on the rate of IGEs at Taboga, and our model overall explained little variance in IGE rates (marginal *R*^*2*^ = 0.00; conditional *R*^*2*^ = 0.010). Specifically, neither cumulative monthly rainfall (β = −0.043, SE = 0.071, z = −0.60, *p* = 0.546) nor maximum monthly temperature (β = −0.027, SE = 0.071, z = −0.38, *p* = 0.704) significantly influenced IGE frequency in our model. As a result, IGE rates at Taboga were consistent across both wet and dry seasons (Fig. 2). In the wet season, the overall rate of IGEs was 0.048 (114 IGEs/2397.92 hours of observation), and in the dry season, the rate was 0.040 (104 IGEs/2586.32 hours of observation).

### Intensity of IGEs at Taboga

**Fig. 3:**
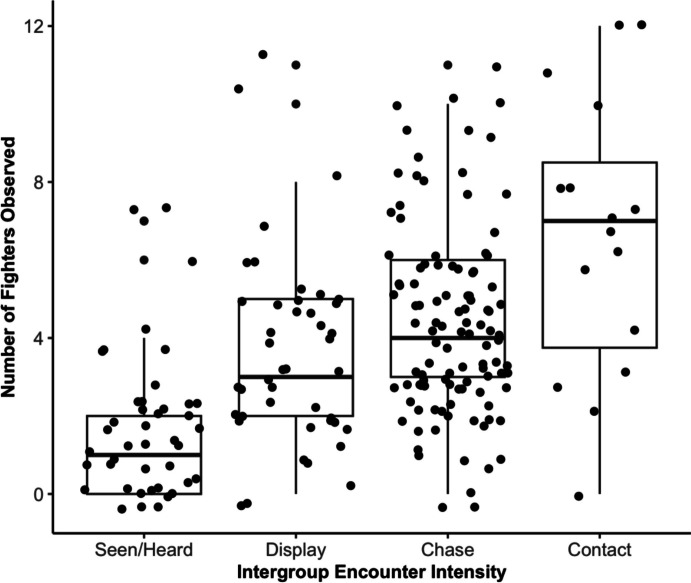
**The number of focal group participants in IGEs of white-faced capuchins (*Cebus imitator*) at Taboga, Guanacaste, Costa Rica (May 2018–May 2022), across increasing intensity of the conflict (from left to right along x axis).** Points represent individual IGEs, boxes indicate the interquartile range (IQR), whiskers extend to values within 1.5 × IQR, and horizontal lines denote medians.

**Fig. 4:**
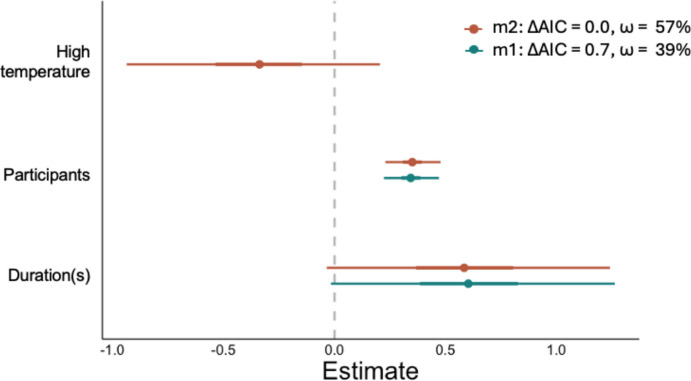
Model-averaged estimates from two top-performing mixed-effects logistic regression models predicting intensity of IGEs. Thick bars represent 50% CI. Thin bars represent 95% CI. Model m2 (ΔAIC = 0.0, weight = 57%) and m1 (ΔAIC = 0.7, weight = 39%) are shown for comparison. Number of participants was a consistent positive predictor in most models we tested. M2, which included high temperature, suggests that white-faced capuchins (*Cebus imitator*) at the Taboga, Guanacaste, Costa Rica (May 2018-May 2022) are less likely to escalate encounters as temperature increase.

Of the 218 IGEs observed from our three main focal groups, 48 (22%) did not result in any aggression between groups (only seeing/hearing another group), 45 (21%) resulted in threats and displays, 107 (49%) escalated to chases between group members, and 18 (8%) resulted in contact aggression between groups (Table S2). Our best fitting ordinal logistic regression model included only two variables: number of participants and the duration of the IGE (df = 6, weight = 57%). For each additional participant observed (β = 0.319, SE = 0.064), the log-odds of being in a higher intensity category increased by 1.42 times, holding the duration constant (Fig. 3). The mean number of participants from the focal group in an IGE was 3.9 individuals (range 1–12) (Table S2). We observed a trend which suggested that longer durations of encounters were associated with higher intensities (β = 0.612, SE = 0.325). The mean duration of an IGE was 26 (range 1–210) min. The next best fitting model (ΔAIC = 0.7, df = 7, weight = 39%) included these same two variables, and *high temperature* (β = −0.335, SE = 0.288) in which higher temperature trended towards an association with lower intensity IGEs (Fig. 4; Table S3). A total of 68% of encounters between groups occurred outside of the core area of the focal group’s home range (Table S2); however, during initial model-screening, predictors describing spatial context (distance to the core of nearest nearest centroid area and distance to water) and group composition (group size and age–sex composition) showed little support and were therefore not retained in the final candidate model set for encounter intensity.

### Participation in IGEs at Taboga

We identified participants in 180 IGEs, as the first IGEs recorded at the site occurred before we began implementing the complete IGE data collection protocol; from these we recorded n=2270 observations of individual participation (yes/no) (Table S2). The model that best fit our data included age, sex, status (alpha: yes/no), distance from the core of the home range, and the interaction between distance to core of the home range and sex as predictors of whether individuals were likely to participate (df = 7, weight = 73%). Capuchins were more likely to participate in IGEs if they were older (β = 1.96, SE = 0.402), male (β = 2.60, SE = 0.386), alpha animals (β = 1.42, SE = 0.514), and closer to the core of the range (β = −1.51, SE = 0.236). The mean age of participants was 11.65 years. The interaction between distance and sex (β = 0.618, SE = 0.309) indicates that males are more likely than females to participate at greater distances, whereas female participation declines as encounters occur farther from the home range core.

**Fig. 5:**
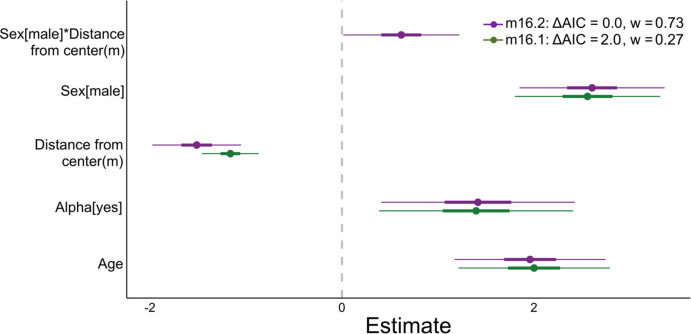
Model-averaged estimates from two top-performing mixed-effects logistic regression models (m16.2 and m16.1) assessing predictors of participation in IGEs. Thick bars represent 50% CI. Thin bars represent 95% CI. Model m16.2 (ΔAIC = 0.0, weight = 73%) included a significant interaction between sex and distance from the centroid of the nearest core area. Alpha status and age were consistently positive predictors across models of wild white-faced capuchins (*Cebus imitator*) at Taboga, Guanacaste, Costa Rica (May 2018–May 2022).

The second-best fit model included the same main fixed effects of age, sex, alpha status, and distance to core of the home range but excluded the interaction effect between distance to core of the range and sex (ΔAIC = 2, df = 6, weight = 27%) (Fig. 5; Table S4). Models that included ecological variables like cumulative rainfall, high temperature, and distance from a water source (m) performed poorly in initial screenings and were not retained our final candidate model set.

**Fig. 6:**
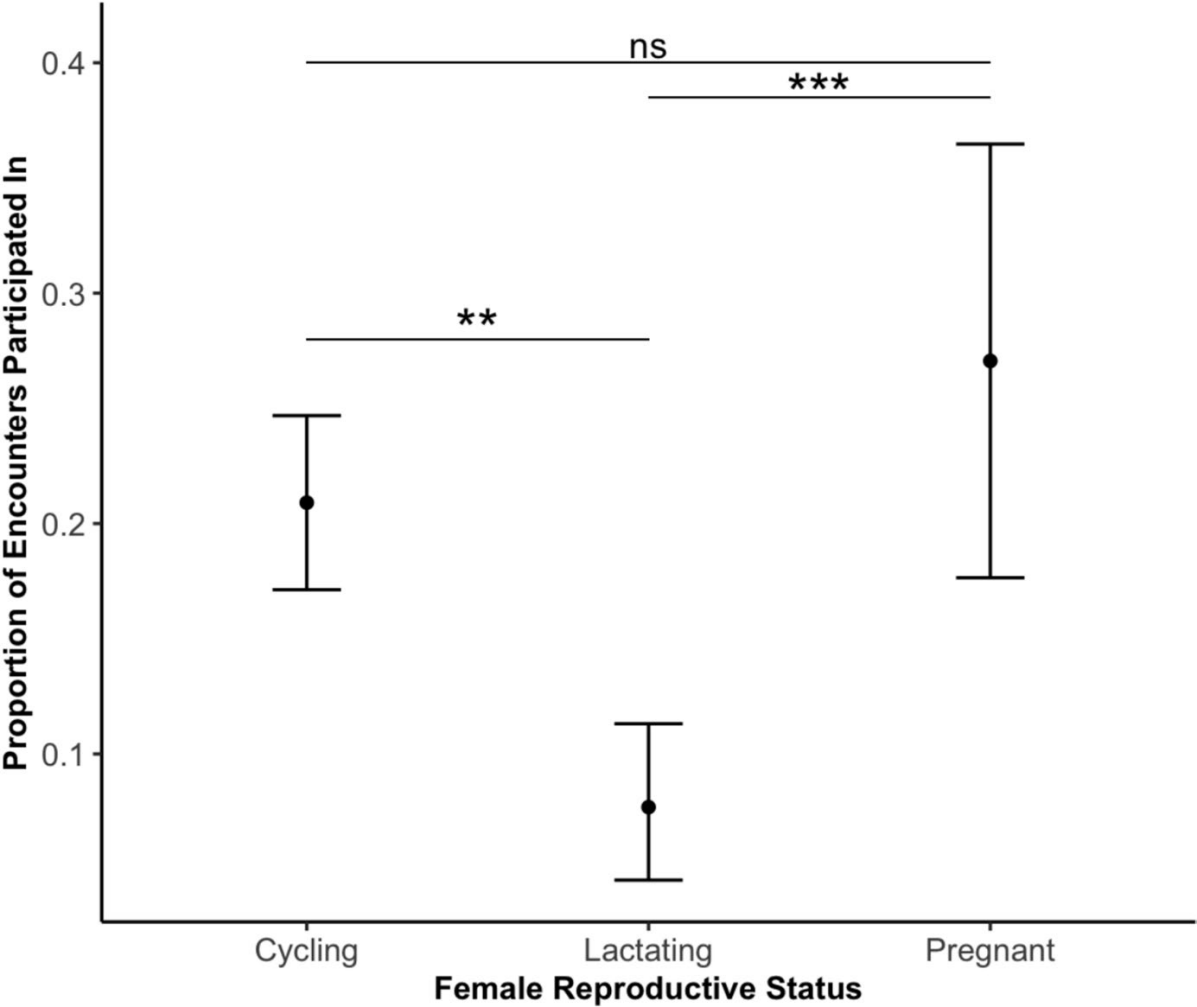
**Proportion of IGEs in which female white-faced capuchins (*Cebus imitator*) at Taboga, Guanacaste, Costa Rica (May 2018–May 2022) participated, shown across reproductive states**. Pregnant females participated significantly more often than lactating females (***p < 0.001), and cycling females participated more than lactating females (*p < 0.01). No significant difference was observed between pregnant and cycling females (ns). Error bars represent 95% confidence intervals.

Overall, females participated in almost half of the IGEs we considered in these analyses (44%, 79/180) (Table S2). Females were significantly less likely to participate in an IGE when they were lactating compared to when they were cycling (β = −0.967, SE = 0.217, *p* = 0.007) or pregnant (*post-hoc Tukey test*, β = −1.500, SE = 0.354, *p* = 0.0001), and there was no significant difference in participation between pregnant and cycling females (β = 0.350, SE = 0.217, *p* = 0.128) (Fig. 6). Reproductive stage explained only a small portion of the overall variation in female participation (marginal R^2^ = 0.075).

## Discussion

Overall, our findings reveal that IGEs at Taboga are frequent with more than half of IGEs categorized as high intensity (i.e., the IGE included chases or contact aggression). The number of participants is strongly associated with IGE intensity, though we do not know in which direction this relationship plays out—with more animals leading to higher intensities or higher-intensity IGEs attracting the participation of more animals. Participation in IGEs largely follows patterns already observed in wild white-faced capuchins with individual characteristics, such as age, sex, and status, emerging as key drivers of participation with male, older, and alpha animals being the most common participants (Crofoot, 2007; Perry, 1996). In our study, ecological variables, such as rainfall, temperature, and distance to a water source, did not seem to hold much explanatory value in models that attempted to explain IGE frequency, intensity, or participation.

### What is the Frequency of IGEs at Taboga, and how does it Compare to Nearby Lower-Density Sites?

Overall, the frequency, intensity, and participation patterns of white-faced capuchins at Taboga are in line with observations from other high-density primate populations (Decellieres *et al.*, 2021; Sugiura *et al.*, 2000; Butynski, 1990). When comparing rates to neighboring field sites, Santa Rosa and Lomas Barbudal, capuchin groups at Taboga exhibited higher frequencies of IGEs (Table I). First, we found that the rate of IGEs at Taboga was 2x higher than that recorded at Santa Rosa and 4x higher than Lomas Barbudal. At Taboga, capuchin density is estimated at 36.24 individuals/km^2^ compared with 11.66 individuals/km^2^ at Santa Rosa and 5.84 individuals/km^2^ at Lomas. The differences in rates of IGEs we present here are in alignment with recorded differences in capuchin populations between those sites, suggesting that the unusually high-density of this population is likely affecting ranging behavior, home range size, and territory overlap leading to more frequency IGEs. For Taboga capuchins, IGEs are an almost daily occurrence.

Consistent with Lomas Barbudal and Santa Rosa, we observed similar rates of IGEs in both wet and dry seasons, with no significant impacts of rainfall or temperature on IGE frequency. This finding suggests that seasonal factors play a minimal role in modulating intergroup interactions in these capuchin populations, supporting the notion again that immediate resources are not the primary driver of these interactions. Anthropogenic disturbances at Taboga, such as irrigation canals providing a constant water supply and year-round access to agricultural crops, may further buffer capuchins from the typical seasonal and ecological stressors observed in other dry tropical forests. In support of this, recent research has shown that Taboga capuchins do not exhibit the common endocrine pattern seen in other tropical mammals, where glucocorticoid levels increase during the dry months when water is scarce and temperatures are high (Beehner *et al.*, 2022). This suggests that capuchins at Taboga may be under higher social than ecological stress compared to less dense populations.

### What Factors Determine the Intensity of IGEs?

We found that IGEs at Taboga are more likely to escalate to aggression than has been reported at nearby sites. While previous studies on white-faced capuchins did not quantify intensity, Perry (1996) states that, “…males occasionally chase other males, and that direct aggression is rare.” At Taboga, we found that chases and direct aggression made up 58% of the intergroups observed for which we rated intensity. Proxies of resource availability, such as cumulative rainfall and distance to the centroid of the nearest core use area (Kernohan *et al.*, 2001), were not related to aggression levels. However, the second-best model included high temperature, which trended towards an association with lower-intensity IGEs—possibly reflecting an adaptive strategy to conserve energy and reduce heat stress. Behavioral strategies, such as resting more and traveling less, have been documented in other tropical primates (*Callithrix jacchus:* De la Fuente *et al.*, 2014. In support of this, we also saw a reduction in distances capuchins travel at Taboga during the dry season. Only the number of participants was a significant predictor of the escalation of IGEs, with each additional participant increasing the odds of higher-intensity interactions by 1.42 times. With a higher density of capuchins, the likelihood of any individual participating is higher.

While theoretical models suggest that IGEs will escalate when 1) the value of the disputed resources is very high or 2) when the resource holding potential (RHP) of groups is evenly matched (Green *et al.*, 2021; Riechert, 1998), neither of these hypotheses can be well assessed. If as some suggest, capuchin IGEs mainly function as an opportunity for males to gain access to mates, it is possible that the relative number of males and females between groups could impact the escalation of IGEs. Unfortunately, we lack this level of data resolution for many of the opponent groups in the dataset. Given the high number of capuchins in the reserve and the frequency of IGEs, it is likely that groups are already somewhat aware of their neighbor’s RHPs. However, there were several demographic shifts during the time of this study (e.g., group fissions, fusions, and takeovers). One of the longest and most aggressive IGEs we observed during the study period occurred in the core of one group’s home range just a month after the group had been overtaken by six males. This intense and prolonged IGE in valuable territory could be evidence that neighbors were testing the new resident males to determine whether they could effectively defend the area. Events such as this could be further investigated to better understand the relationship between abrupt changes in group composition and intergroup aggression.

Payoff asymmetries, in which two parties in a conflict or competition derive unequal benefits or costs from a particular outcome, can also impact intensity in intergroup competitions. For example, if one group frequently uses a specific water source or feeding area while the other group rarely does, the first group has a higher "payoff" for maintaining access, which might lead to more aggressive or determined defense efforts (Riechert, 1998). In alignment with this, increased aggression is common in other primate IGEs occurring in core territory (Kitchen *et al.*, 2004; Morrison *et al.*, 2020) and aggression between blue monkey groups increased in IGEs when groups’ use of the area in question was more similar over the previous six months (Roth & Cords, 2016). These findings support the idea that IGEs can become more aggressive when opposing groups use a site to a similar extent or when the site is equally central to both groups' home ranges, likely because such areas are valued equally, reducing the likelihood of retreat and increasing aggression. At Taboga, high levels of territory overlap may amplify these dynamics, as shared resource areas create more opportunities to meet and compete for equally contested spaces.

While the above theories can be instructive for research on IGEs, many of these ideas were developed in the context of competition between individual organisms, while competition between groups is considerably more complex as it requires the cooperation of multiple individuals. In group living species, competitive ability and RHP can be undermined by the collective action problem (Beehner & Kitchen, 2007; Willems *et al.*, 2013), when collective action results in public goods and the interests of group members are not aligned. We found that greater participation and higher IGE intensity co-occur, indicating that social dynamics may play an important role in shaping conflicts. This pattern could reflect audience effects (Cheney & Seyfarth, 1990) and behavioral contagion (Preston & De Waal, 2002), whereby the presence and actions of conspecifics increase individual propensity to engage, or in the other direction, numerical assessment and risk dilution processes that promote escalation as the number of in-group participants increases.

Although we cannot determine the direction of causality from these data, and the observed association may reflect the influence of unmeasured variables, the pattern is consistent with broader comparative evidence highlighting the importance of social influence in capuchins. There is support for audience effects and behavioral contagion in capuchins, with evidence that males are more likely to participate in intergroup aggression when another male is present, particularly if that male responds aggressively (Meunier *et al.*, 2012). More broadly, our findings are consistent with a meta-analysis of 138 primate species showing that the intensity of IGEs is more strongly impacted by social factors than by environmental conditions (Willems & van Schaik, 2015). In capuchin IGEs, whether increased participation facilitates escalation or escalating interactions draw in more participants, both scenarios highlight how social influence from peers might serve as a form of motivation or reinforcement to join and sustain higher-intensity interactions, aligning individual decisions with group interests.

### What Drives Variation in Individual Participation During IGEs?

The likelihood of participating in an IGE was significantly influenced by individual characteristics, with older, male, and alpha individuals more likely to engage aligning with past research into capuchin participation (Perry, 1996: Van Belle & Scarry, 2015). However, at Taboga, females participated in 44% of intergroups (79/180), a high percentage in contrast to Lomas Barbudal, where females only participated in 11% (5/44) of IGEs (Perry, 1996), but consistent with Santa Rosa where females only participated slightly less than males (Rose, 1994). Proximity to the nearest home range core centroid also influenced participation, with involvement decreasing farther from a core area. Core areas (50% KDEs) represent regions of concentrated space use that groups have more detailed knowledge of and are often, though not necessarily, associated with key resources such as feeding or resting sites (Kernohan *et al.*, 2001). Interestingly, males were more likely than females to participate in encounters farther from the core, while closer proximity appeared to encourage higher involvement of the philopatric sex, potentially reflecting greater female investment in defending central, high-use, and familiar areas. While more participants were associated with greater IGE intensity, distance from the core did not predict the escalation of aggression. Given that home ranges at Taboga (mean = ~1.2 km^2^, range = ~0.7**–**1.5 km^2^) are smaller than those at Lomas Barbudal (mean = ~2.5 km^2^, range = ~0.5**–**7.5 km^2^) (Jacobson *et al.*, 2024), IGEs at Taboga presumably occur closer to core areas of group territories. As a result, females at Taboga may be more likely to participate in these encounters due to their proximity to the group's central resources and social hub. In contrast, females at other sites with larger home ranges may be less involved in IGEs because the encounters take place farther from these valuable core areas.

We also observed that female participation in IGEs was influenced by reproductive stage. Lactating females were significantly less likely to engage in IGEs than cycling or pregnant females, likely due to high risk of injury or death for offspring. This is of interest because work in tufted capuchins (*Sapajus nigritus*) demonstrates that dependent offspring did not inhibit female participation in intergroups (Scarry, 2012). Infanticide is a risk for both tufted and white-faced capuchins (*Cebus imitator*) (Ramírez-Llorens *et al.*, 2008; Fedigan *et al.*, 2021), although variation in frequency of infanticide and its association with IGEs may shape female strategies. At Santa Rosa, where infanticide occurs frequently, females do not exhibit male-mediated pregnancy loss (Petersdorf *et al.*, 2025), suggesting that cautious engagement during IGEs and alloparenting may function as strategies to mitigate this risk. In tufted capuchins, the need to maintain access to core areas while managing the high energetic burden of nursing might outweigh the risks of participating despite the threat of infanticide. At Taboga, IGEs followed by alpha male takeovers and infanticide are relatively common, suggesting that the risk of infanticide linked to IGEs may reduce participation by females with dependents. However, reproductive stage accounted for a small amount of variation in female participation, suggesting that other factors, such as rank, social alliances or individual costs, play more critical roles in shaping female behavior during IGEs.

Our study adds to the growing body of literature on capuchin social dynamics, highlighting the complex interplay between environmental, demographic, and social factors in shaping intergroup competition. Rainfall as a proxy for season did not significantly predict IGE intensity or frequency, which we interpret as an indication of site richness and the central role of social dynamics in shaping conflict. However, it is possible that our ecological measures were insufficiently precise to detect subtle impacts, and so we encourage work incorporating phenology data and specific food resources, such as the invasive neem tree (*Azadirachta indica)* which may elucidate more patterns of intergroup competition. The observed relationship between participant numbers and aggression intensity warrants further investigation to decipher the causal direction of this relationship and suggests that social coordination—potentially driven by audience effects and social facilitation, or alternatively by numerical assessment and risk dilution—plays a key role in overcoming collective action challenges during group defense. Future work using fine-scale temporal analyses of synchronized video and audio recordings of IGEs could help to elucidate these mechanisms. Overall, males participated in IGEs more frequently than females; however, female participation at Taboga was higher than that reported at nearby sites and varied with proximity to core use areas and reproductive stage. A valuable next step would be to test whether variation in female participation tracks infanticide risk, across species and study sites. Finally, research should investigate the impacts of frequent between-group aggression on the monkeys at Taboga, especially in terms of stress profiles, group cohesion, and demographic shifts, to better understand the evolutionary significance of these encounters, particularly in capuchin populations that inhabit rapidly shrinking territories.

## Supplementary Information

Below is the link to the electronic supplementary material.

Supplementary file1 (DOCX 4718 KB)
