# Supplementary material for: Intergroup Encounters Among Wild White-Faced Capuchins (Cebus imitator) at a Densely Populated Field Site: Insights into Frequency, Intensity, and Participation
Source: Int J Primatol. 2026 Apr 6;47(3):616–40. doi: 10.1007/s10764-026-00548-z (PMC13323443; doi:10.1007/s10764-026-00548-z)
Supplement: Supplementary file 1 — Supplementary file1 (DOCX 4718 KB) [file 10764_2026_548_MOESM1_ESM.docx]

**S1.** Home range area (95% kernel density estimation) and core areas (50% kernel density estimation) of three groups of white-faced capuchins (*Cebus imitator*) at the Taboga Forest Reserve, Guanacaste, Costa Rica (May 2018-May 2022), Tenori, Palmas, and Mesas group with IGEs, water sources, and core area centroids indicated.

**
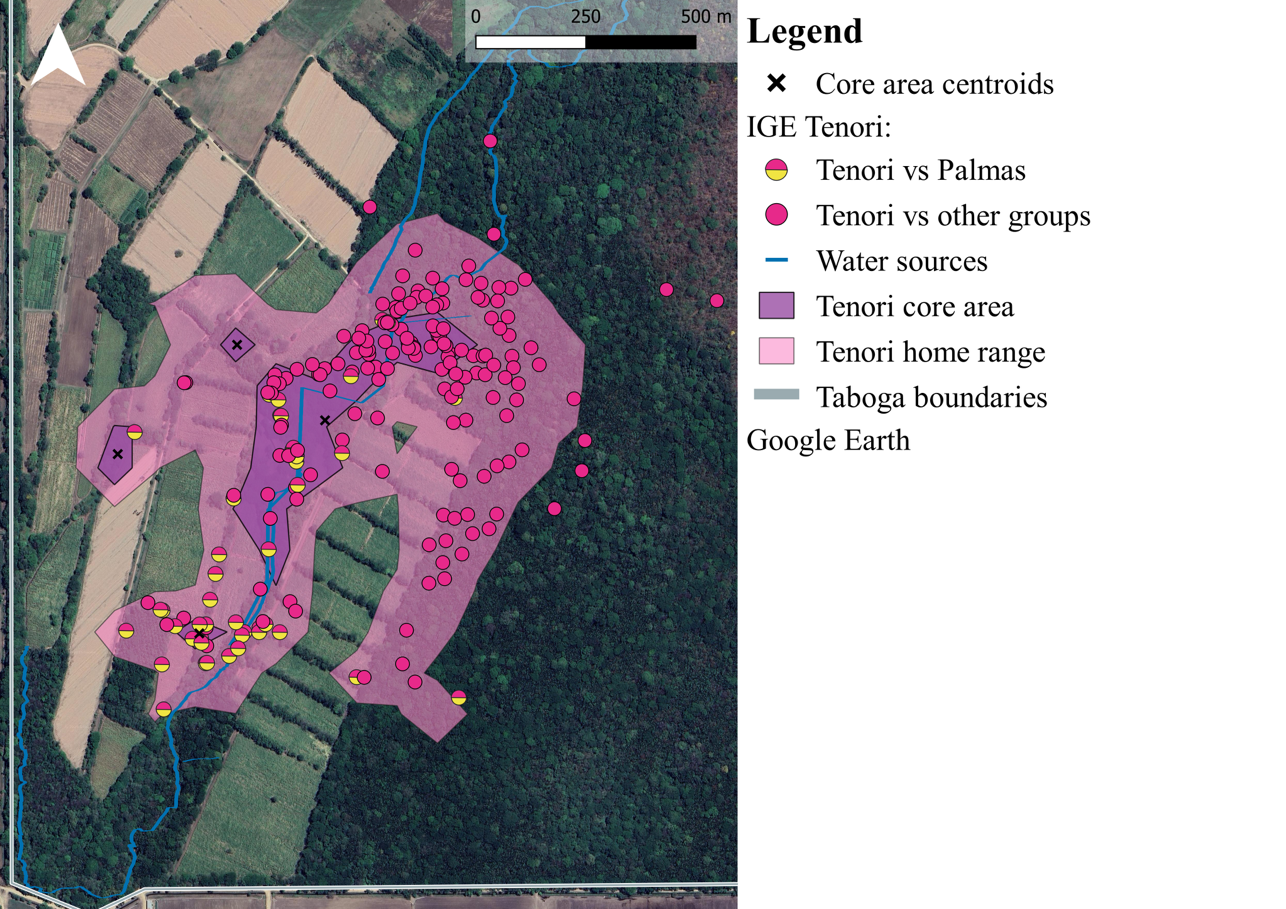
**

**
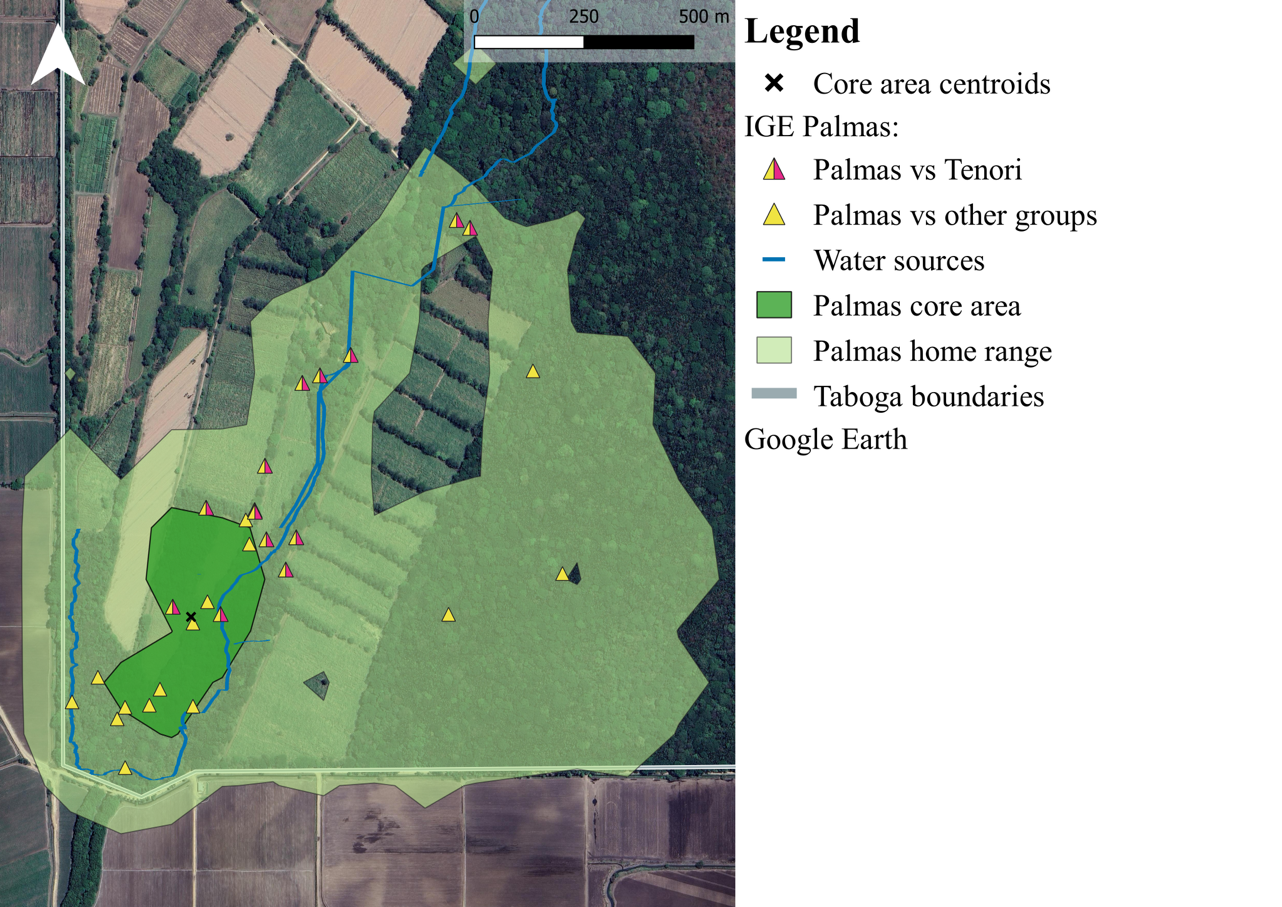
**

**
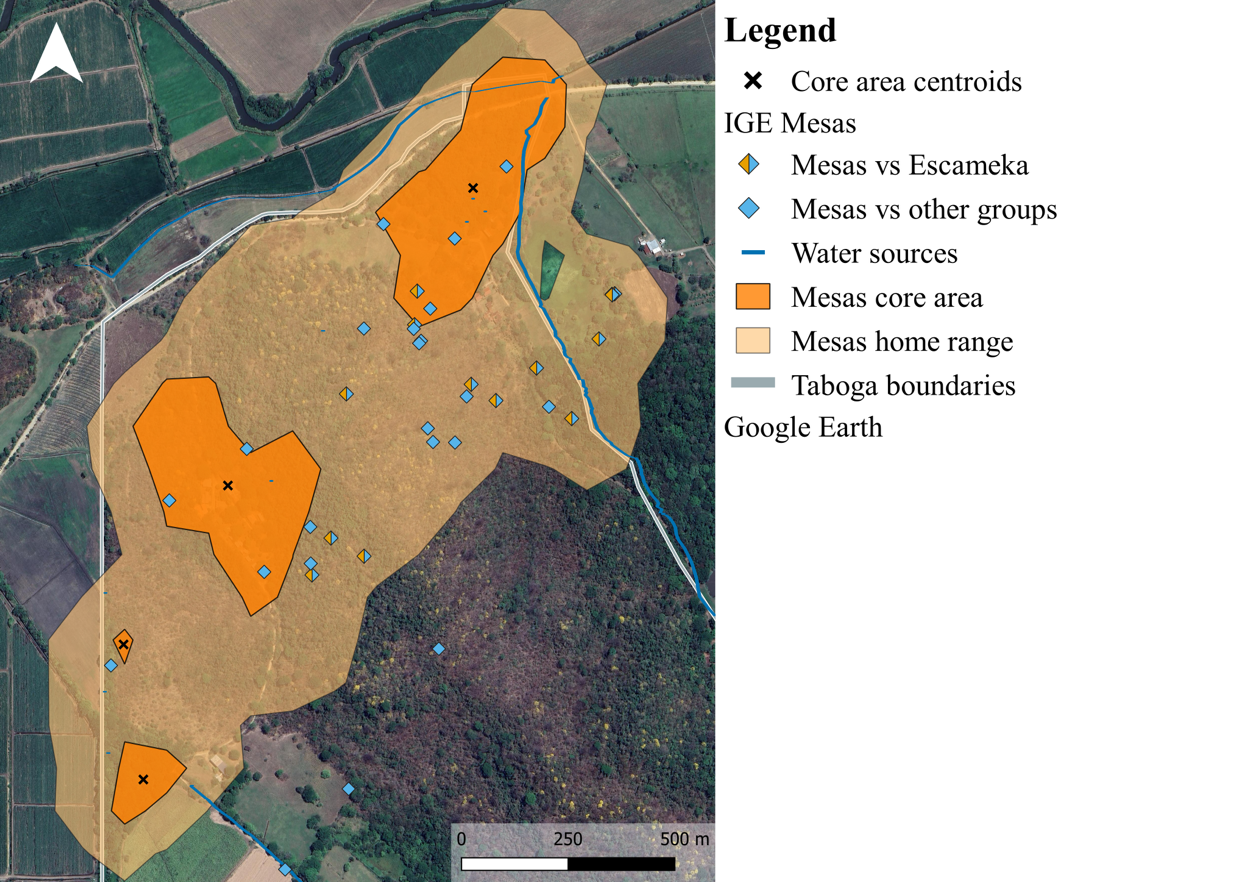
**

**Home range and core areas.** Analyses were conducted in R version 4.1.2 (R Core Team, 2021) using the package *adehabitatHR* (v. 0.4.21; Calenge, 2011). Using GPS ranging data, we estimated home ranges (95% kernel density estimation, KDE) and core areas (50% KDE) for the Tenori, Mesas, and Palmas capuchin groups. Kernel utilization distributions were calculated using the function kernelUD() with the reference bandwidth method (href), pooling all available GPS locations collected for each group across the study period (2018–2022; Tenori: *n* = 538,327 points; Mesas: *n* = 287,050 points; Palmas: *n* = 151,887 points). Home range and core area isopleths (95% and 50%, respectively) were then extracted using the function getverticeshr().

Spatial data handling and distance calculations were performed using the packages *sp* (v. 1.4-6), *rgdal* (v. 1.5-28), *rgeos* (v. 0.5-9), *raster* (v. 3.5-15), and *sf* (v. 1.0-7). Spatial outputs were exported as ESRI shapefiles and visualized in QGIS (v. 3.16.16; QGIS Development Team, 2021).

Because core areas may consist of multiple disjoint polygons, we calculated centroids from the full 50% KDE core area of each group and used these centroids as spatial references. Distance from the center of the home range has been shown to influence intergroup encounter outcomes in white-faced capuchins (Crofoot et al., 2008). Accordingly, for each observed intergroup encounter (IGE), we used the GPS point recorded at the start of the encounter to calculate (i) the Euclidean distance to the centroid of the nearest core area of the focal group (*dist.centroid*) and (ii) the distance to the nearest water source. These variables were used to examine their effects on encounter intensity (Question 2) and individual participation (Question 3).

**S2.** Descriptive statistics of Intergroup Encounters at Taboga

| **Total IGEs**  **n=218** | | **Mean ± SD** | **Median** | **Range** |  |
| --- | --- | --- | --- | --- | --- |
|  | Participants | 3.86 ± 2.63 | 3 | 0-15 |  |
|  | Duration (min) | 26.0 ± 27.04 | 19 | 0-210 |  |
|  | Distance from centroid (m) | 303.07 ± 176.5756 | 299.37 | 14.72-1090.04 | |
|  | Distance from water (m) | 118.6 ± 118.85 | 71.99 | 1.10 – 495.47 | |
|  |  | **Seen/Heard** | **Display** | **Chase** | **Contact** |
|  | Intensity Level (count/%) | 48 (22%) | 45 (21%) | 107 (49%) | 18 (8%) |
| **Participation**  **n=180** | | **Mean ± SD** | **Median** | **Range** |  |
|  | Participant Age (years) | 11.7 ± 5.6 | 5.86 | 0.1-28.7 |  |
|  | Males (# participants per IGE) | 3.65 ± 2.41 | 3 | 1-15 |  |
|  | Females (# participants per IGE) | 0.97 ± 1.45 | 0 | 0-6 |  |
|  |  |  |  |  |  |
|  |  | **% of IGEs participated in** | | **# of IGES/180** |  |
|  | Males (total) | 100% | | 180 |  |
|  | *Adult* | 95% | | 171 |  |
|  | *Subadult* | 83.3% | | 150 |  |
|  | *Juvenile* | 32.8% | | 59 |  |
|  | Females (total) | 43.9% | | 79 |  |
|  | *Adults* | 38.3% | | 69 |  |
|  | *Juveniles* | 20% | | 36 |  |

**S3:** Comparison of generalized linear mixed model results for intensity level of IGEs in white-faced capuchins (*Cebus imitator*) at the Taboga Forest Reserve, Guanacaste, Costa Rica (May 2018-May 2022), using AICc. Glm_2 holds most of the model weight compared to the other models.

| **model** | **predictor** | **estimate** | **std.error** | | **ΔAICc** | **df** | **weight** |
| --- | --- | --- | --- | --- | --- | --- | --- |
| glm_2 | Participants | 0.32 | | 0.06 | 0.0 | 6 | 57 |
|  | Duration | 0.61 | 0.33 | |  |  |  |
| glm_1 | Participants | 0.33 | 0.06 | | 0.7 | 7 | 39 |
|  | Duration | 0.59 | 0.32 | |  |  |  |
|  | High temperature | -0.33 | 0.29 | |  |  |  |
| glm_3 | Participants | 0.32 | 0.06 | | 5.3 | 10 | 4 |
|  | Duration | 0.59 | 0.33 | |  |  |  |
|  | High temperature | -0.36 | 0.29 | |  |  |  |
|  | Cumulative rain | 0.11 | 0.32 | |  |  |  |
|  | Distance to core | -0.38 | 0.33 | |  |  |  |
|  | Distance to water | 0.35 | 0.33 | |  |  |  |

**S4.** Comparison of generalized linear mixed model results for participation in white-faced capuchin (*Cebus imitator*) IGEs at the Taboga Forest Reserve, Guanacaste, Costa Rica (May 2018-May 2022), using AICc. Glmm_16.2 holds most of the model weight compared to the other models.

|  | **model** | **predictor** | **estimate** | **std.error** | | **ΔAICc** | **df** | **weight** |
| --- | --- | --- | --- | --- | --- | --- | --- | --- |
|  | glmm_16.2 | Age | 1.96 | 0.40 | | 0.0 | 7 | 73 |
|  |  | Sex | 2.61 | 0.39 | |  |  |  |
|  |  | Rank | 1.42 | 0.51 | |  |  |  |
|  |  | dist.center | -1.51 | 0.24 | |  |  |  |
|  |  | Dist.center*Sex | 0.62 | 0.31 | |  |  |  |
|  | glmm_16.1 | Age | 2.00 | 0.40 | | 2.0 | 6 | 27 |
|  |  | Sex | 2.56 | 0.39 | |  |  |  |
|  |  | Rank | 1.40 | 0.52 | |  |  |  |
|  |  | dist.center | -1.16 | 0.15 | |  |  |  |
| glmm_16.0 | | Age | 2.12 | | 0.40 | 62.6 | 5 | <0.001 |
|  | | Sex | 2.05 | 0.39 | |  |  |  |
|  | | Rank | 1.37 | 0.51 | |  |  |  |
